# Assessment of regional networks on nutrition in South Asia: a multi-methods study

**DOI:** 10.1186/s12889-022-12585-3

**Published:** 2022-01-26

**Authors:** Harriet Torlesse, Jenny Ruducha, Carlyn Mann, Zivai Murira

**Affiliations:** 1 UNICEF Regional Office for South Asia, Leknath Marg, Kathmandu, 44600 Nepal; 2 Braintree Global Health, 4322 West Point Place, Vancouver, BC V6R 4M9 Canada

**Keywords:** Organizational network analysis, Qualitative research, Regional networks, Social networks, South Asia, Undernutrition

## Abstract

**Background:**

Many national and international organizations are working to improve maternal and child nutrition in countries with high malnutrition prevalence and burdens. While there has been progress in strengthening multi-organizational networks on nutrition at country and global levels, the regional level has received much less attention. We conducted a study to 1) determine the existing relationships and levels of engagement between international organizations working to improve nutrition at the regional level or in at least two countries in South Asia; and 2) examine the experiences and perspectives of international organizations on regional-level communication, coordination or collaboration on nutrition in South Asia.

**Methods:**

A mixed methods approach involving organizational network analysis (ONA) and semi-structured interviews was used to develop an understanding of the existing network and relationships between international organizations working on nutrition in South Asia. ONA data from 43 international organizations was analysed using a social network analysis software (UCINET) to systematically quantify and visualize the patterns of relationships between organizations.

**Results:**

We found a high degree of connectivity between most of the international organizations in South Asia, but there were gaps between the many organizations that knew each other and the work they did together regionally on nutrition. Most organizations worked together only ‘rarely’ or ‘sometimes’ on nutrition at the regional level and high-intensity (collaborative) working relationships were uncommon. Organizations of the same type tended to cluster together, and a small number of UN agencies and multilateral organizations were central brokers in the nutrition working relationships. Perceived constraints to the nutrition working relationships included organizations’ agenda and mandate, threats to visibility and branding, human and financial resources, history, trust and power relations with other organizations, absence of a regional network for cooperation, and donor expectations. There was high demand to remedy this situation and to put network mechanisms in place to strengthen communication, coordination and collaboration on nutrition.

**Conclusions:**

Opportunities are being missed for organizations to work together on nutrition at the regional level in South Asia. The effectiveness of regional nutrition networks in influencing policy or programme decisions and resources for nutrition at country level should be explored.

## Background

Many national and international organizations are supporting efforts to improve maternal and child nutrition in countries with high malnutrition prevalence and burdens, including United Nations (UN) agencies, multilateral and bilateral donors, civil society organizations, philanthropic foundations and businesses. These organizations work across one or more sectors, at various levels (subnational, national, regional and/or global), and often share similar goals, objectives, strategies and interventions with other organizations. A multi-stakeholder and multi-sector response to nutrition is needed because the multiple determinants of malnutrition require a range of actors to intervene across all the sectors that influence nutrition outcomes [1–4].

In 2008, international organizations came under scrutiny for failing to ensure complementary, mutually reinforcing, and well-designed solutions to nutrition challenges at country level [5]. A review of evaluations, combined with observations from key informant interviews, found that weak coordination, collaboration, and linkages with countries were among the problems reducing their collective effectiveness [5]. Since then, the global nutrition landscape has been transformed through initiatives such as the global Scaling Up Nutrition (SUN) Movement, which helped to strengthen the coordination architecture on nutrition and guide countries on aligning stakeholders around commonly agreed national nutrition priorities [6, 7]. Countries that join the SUN Movement are encouraged to form multi-sector and multi-stakeholder coordination mechanisms to oversee the design and implementation of multi-sector nutrition strategies and plans [8]. Countries may also form single-stakeholder groups to coordinate actions at country level (donors, United Nations, civil society and business), which can draw support from corresponding SUN stakeholder networks at global level.

While there has been progress in strengthening coordination on maternal and child nutrition between organizations at the country and global levels, the regional level has received much less attention. The SUN Movement has not actively pursued stakeholder platforms or networks at the regional level [9], despite the close geographic and working proximity of regional organizations to countries and their potential to align resources, amplify nutrition-related advocacy, provide policy and programme guidance, develop capacity and foster cross-country learning on issues that are not confined to a single country.

In the South Asia region (Afghanistan, Bangladesh, Bhutan, India, Maldives, Nepal, Pakistan and Sri Lanka), there are a variety of organizations that are working in multiple countries or at the regional level to bring about sustainable change in maternal and child nutrition. However, little is known about how these organizations interact with one another to support efforts to improve maternal and child nutrition in the region. As of 2018, there was no regional network that brought these organizations together to share information, coordinate or collaborate on nutrition even though the region’s eight countries are home to over half of the world’s children with wasting (children who weigh too little for their height) and 40% of the world’s children with stunting (children who are too short for their age) [10].

Concerned that international organizations could be missing opportunities to better harness and align goals and resources to tackle South Asia’s nutrition challenges, we conducted a study to 1) determine the existing relationships and levels of engagement between international organizations working at the regional level or in at least two countries in South Asia to improve maternal and child nutrition in South Asia, and 2) examine the experiences and perspectives of international organizations on regional-level interactions on maternal and child nutrition in South Asia.

## Methods

### Study design

A mixed methods approach, involving organizational network analysis (ONA) and semi-structured interviews, was used to develop an understanding of the existing network and relationships between regional-level organizations working on nutrition in South Asia. ONA, which is also referred to as Social Network Analysis or Network Analysis, is a method that systematically quantifies and visualizes the patterns of relationships within or between organizations [11]. The term ‘network’ is used to describe multi-organizational relationships, which can take different forms, such as alliances, associations, collaborations and partnerships. ONA examines how the strength, frequency and nature of interactions between organizations influences the dynamics and performance of the overall network. ONA has been used previously to study relationships within multisectoral and multi-stakeholder networks, including on health and nutrition in South Asia [12–14]. The semi-structured interviews explored the organizations’ perspectives and experiences on working relationships between organizations at regional level to provide further context and possible explanations for the ONA findings.

### Identification of the study sample

The study sample included organizations working to improve nutrition across a range of sectors through either nutrition-specific or nutrition-sensitive approaches in at least two South Asian countries or at the regional level. These organizations were identified by the lead researcher and key informants in the United Nations Children’s Fund (UNICEF) Regional Office for South Asia (ROSA) and other regional organizations using a range of techniques, including key informant recall, snowball sampling and internet searches to expand beyond the set of well-known actors. The final list was comprised of 50 organizations, including United Nations (UN) agencies, bilateral and multilateral donors, non-government organizations (NGOs), academic/research institutions, an intergovernmental organization, foundations, and networks. The organizations are listed in Table 1, together with the acronyms that are used hereafter in this article and represent a wide range of stakeholders and nutrition-relevant sectors. All organizations were asked to participate in the ONA and a subset of 26 organizations with a mandate on nutrition, as assessed using available information, were selected for the semi-structured interviews.

**Table 1:** Organizations interviewed for the ONA and qualitative interviews

Name of organization	Acronym	Type	ONA	Qualitative
Aga Khan Development Network	AKDN	Foundation	*	
Aga Khan University, Centre for Excellence of Women and Children	AKU-CWC	Academic/research	*	
Alive & Thrive, South Asia Region	A&T	NGO	*	*
Asia Civil Societies Group for the Scaling Up Nutrition Movement	CivilSUN	Network	*	*
Asia Regional Network on Early Childhood	ARNEC	Network	*	
Asian Development Bank	ADB	Multilateral	*	
Asian Farmers Association	AFA	Network	NR	
Asian NGO Coalition	ANGOC	Network	*	
Bangladesh Rural Agricultural Committee	BRAC	NGO	*	*
Bill & Melinda Gates Foundation	BMGF	Foundation	*	*
Children's Investment Fund Foundation	CIFF	Foundation	NR	*
Consortium of South Asian Think Tanks	COSATT	Network	*	
Department for International Development, United Kingdom	DFID	Bilateral	*	
Emergency Nutrition Network, Asia Region	ENN	Network	*	*
European Community Humanitarian Office, Regional Office for East, South East Asia and Pacific region	ECHO	Bilateral	*	
European Union International Cooperation and Development	EUDEVCO	Bilateral	*	
Food and Agriculture Organization, Asia–Pacific Region	FAO	UN	*	*
Foreign Correspondents Club of South Asia	FCC-South Asia	Academic/research	*	
Global Alliance for Improved Nutrition	GAIN	NGO	*	*
Helen Keller International, Asia Pacific Region	HKI	NGO	*	*
HomeNet, South Asia	HomeNet	Network	*	
International Baby Food Action Network, South Asia	IBFAN	Network	*	*
International Food Policy Research Institute, South Asia	IFPRI	Academic/research	*	*
International Labor Association Decent Work Technical Support Team	ILO_DWT	UN	*	
Iodine Global Network, South Asia	IGN	NGO	*	*
Lakshmi Mittal South Asia Institute, Harvard University	SAI	Academic/research	NR	
Leveraging Agriculture for Nutrition in South Asia	LANSA	Network	*	*
Nutrition International, Asia Region	NI	NGO	*	*
Save the Children, Asia Region	SAVE	NGO	*	*
South Asia Food and Nutrition Security Initiative (World Bank project)	SAFANSI	Multilateral	*	*
South Asia Foundation	SAF	Foundation	NR	
South Asia Infant Feeding Research Network	SAIFRN	Network	*	
South Asia Watch on Trade, Economics and Environment	SAWTEE	Network	*	*
South Asian Association for Regional Cooperation	SAARC	Intergovernmental body	*	*
South Asian Policy Leadership for Improved Nutrition and Growth	SAPLING	Academic/research	*	*
South Asian Women Development Forum	SAWDF	Network	NR	
South Asia Women’s Network	SWAN	Network	NR	*
Sustainable Development Solutions Network at TERI University	SDSN	NGO	*	
Tata-Cornell Institute for Agriculture and Nutrition	TCI	Academic/research	*	
Tata Trusts, India	Tata	Foundation	*	*
UNICEF Regional Office for South Asia	UNICEF ROSA	UN	*	*
United Nations Fund for Population, Asia and Pacific	UNFPA	UN	*	
US Agency for International Development	USAID	Bilateral	*	
Water Aid, South Asia	Water Aid	NGO	*	
Water Supply and Sanitation Collaborative Council	WSSCC	UN	*	
White Ribbon Alliance	WRA	Network	*	
World Bank	WB	Multilateral	*	*
World Food Programme, Asia Bureau	WFP	UN	*	*
World Health Organization, Eastern Mediterranean Regional Office	EMRO	UN	*	*
World Health Organization, South East Asia Regional Office	SEARO	UN	*	*

*NGO* non-nongovernment organization, *REC* regional economic commission, *UN* United Nations, *NR* no response

The respondents included senior managers or technical leads with 77% having worked with their current organization for 3 or more years. Respondents were responsible for nutrition portfolios and knowledgeable about the relationships of their own organization with other organizations working on nutrition. However, some respondents engaged other colleagues to provide more information on specific qualitative questions. For organizations without a regional office in South Asia or other sub-regions of Asia, the individual responsible for nutrition and/or South Asia in a headquarter-level or country-level office was interviewed.

### Data collection

An on-line ONA questionnaire was developed using the Survey Monkey platform and sent to 50 organizations in March 2018 to gather information on the frequency, nature and intensity of relationships between organizations in the last two years prior to the survey. The ONA questionnaire included a series of questions about the relationship of the responding organization with every other organization in the study sample. The first ONA question established whether there was a relationship between two organizations for any purpose, including but not limited to nutrition. If a bilateral relationship was confirmed, a second question was asked about the intensity of the relationship between the two organizations. The three levels of intensity were defined, starting with communication, followed by coordination and collaboration, based on organizational frameworks of relationship strength [15, 16]. Next, questions were asked to establish whether the two organizations had working relationships on nutrition, hereafter referred to as “nutrition working relationships”: 1) nutrition-related policies, legislation, strategies or plans; 2) capacity development on nutrition; 3) knowledge management on nutrition; and 4) implementation of nutrition-related interventions. The responses were recorded as a frequency of contact using a Likert scale (not at all, rarely, sometimes, often and very often).

Semi-structured interviews were conducted by an external investigator engaged by the UNICEF ROSA in March and April 2018. Interviews were conducted in-person in Kathmandu, Nepal, and New Delhi, India, or remotely using Skype for individuals who were based elsewhere or not available during the time of the country visits. A semi-structured qualitative interview guide with open-ended questions was used during the interviews and explored a range of themes based on adaptation of the Integrative Framework for Collaborative Governance [17] and Framework for Cross Sectoral Collaboration [18]. Multiple sub-questions and probes were used to guide the discussion and triangulate perspectives and experiences. The duration of each interview ranged from 45 to 90 min and were recorded after obtaining informed consent from the respondent.

### Data analysis

ONA data from the Survey Monkey questionnaires were entered into Excel files and matrices were constructed for each ONA measure (see Table 2 for a list and definitions of all ONA measures). The data was analysed using UCINET software Version 6 [19] and visual plots were created using NetDraw [20].

**Table 2:** Definitions of Network Measures (Freeman, 1978; Hanneman & Riddle, 2005)

**Network level ties**
*Density* is defined as the ratio of ties divided by the number of possible ties. A network’s density may influence the speed at which information diffuses among the nodes/organizations and the extent to which actors have high levels of social capital and/or social constraint
*Centralization* is an expression of how tightly the network structure is organized around its most central point. The general procedure involved in any measure of graph centralization is to look at the differences between the centrality scores of the most central point and those of all other points. Centralization is the ratio of the actual sum of differences to the maximum possible sum of differences
**Node or individual organizational ties**
*Normalized degree centrality* measures adjacent links to or from an organization divided by the possible number of links, expressed as a percentage, and reflects the potential power of direct relationships. These direct links reduce the reliance on intermediaries to access information or resources. More connections are generally considered better than fewer connections
*Normalized betweeness centrality* measures the extent to which organizations fall between pairs of other organizations on the shortest paths (geodesics) connecting them adjusting for the number of pairs in the network. This measure represents potential mediation or flow of information or resources between organizations in the network. It is used to assess the power in networks, as an organization may control the flow of information and potential resources, thereby increasing dependence of others who are not directly connected in the network
**Relationship level ties**
*Intensity* describes the level of interaction between different organizations. In this study, the level of interactions between pairs of organizations was classified as communication (sharing of information between organizations), coordination (organizations implement actions independently, based on shared goals, plans and/or information) and collaboration (organizations jointly create and/or execute actions)
*Multiplexity* is a measure that describes multiple relationships among the same set of organizations. In this study, four types of working relationships were examined: (a) policy, legislation, strategies and plans; (b) capacity development; (c) knowledge management; and (d) implementation. The multiplexity score ranged from 1 to 4, depending on the number of confirmed working relationships

Two sets of ONA plots were created to visualize the relationships between organizations in the regional network. The first set examined the overall network and relationships between organizations on any purpose over the past two years. This included the existence of any type of relationship between pairs of organizations and the intensity of these relationships (communication, coordination or collaboration). The second set analysed the frequency of interactions on nutrition working relationships (nutrition-related policies, legislation and strategies; nutrition-related capacity development; support for implementation of nutrition-related interventions; and nutrition-related knowledge management). This information was then used to create a multiplexity measure, which assessed the strength of the relationship by determining the total number of nutrition working relationships (range one to four) that organizations were working on together.

Because the reported relationships were based on the key informants’ perceptions, a confirmation process was used to improve the reliability of the data. A confirmed relationship is formed when two organizations both acknowledge a relationship with each other. For example, organization A must report a relationship with organization B and organization B must report a relationship with organization A to record a confirmed relationship. For responses with multiple possible options (intensity of relationship and frequency of contact), a minimum confirmation process was used. For example, if organization A reported that it ‘often’ had contact with organization B on knowledge management, whereas organization B indicated it had contact with organization A ‘sometimes’, then the confirmed frequency of contact was recorded as ‘sometimes’.

The plots were constructed with nodes and lines that were shaped and/or coloured to represent characteristics of interest. The node shapes represent the number of South Asian countries in which the organizations operated, and the node colours represent the types of organizations. The size of the nodes was adjusted for betweenness centrality. The line colours denote either the intensity of the working relationship, the frequency of contact or the number of nutrition working relationships.

Qualitative data from the semi-structured interviews were abstracted into a predesigned matrix that was organized by key themes of enquiry. Data were cleaned, and any inconsistencies were double-checked with the audio recordings. Differences in views or range of perceptions were noted and further explored to understand the dynamics in the responses. The information was then synthesized across major domains and sub-domains that included 1) experiences of regional networks in working together on nutrition in South Asia; 2) perspectives on factors affecting regional-level communication, coordination or collaboration on nutrition; and 3) identification of processes and demand for strengthening of networks and overcoming constraints to collaboration on regional nutrition goals and their execution.

## Results

### ONA: network linkages and dynamics

The ONA questionnaire was completed by 43 (86%) of 50 targeted organizations (Table 1). The responding organizations included UN agencies (19%), donors (16%), NGOs (21%), academic and research institutes (9%), an intergovernmental organization (2%), foundations (9%), and networks (23%). Most of the organizations worked in Bangladesh (76%), followed by India (74%), Pakistan (68%), Nepal (64%), Sri Lanka (52%), Afghanistan (46%), Bhutan (36%) and the Maldives (34%). Twelve organizations (24%) worked in all eight South Asian countries.

ONA measurements were used to explore how organizations interact in the overall regional network and across each of the four nutrition working relationships in this study. These measurements include density (extent to which all potential connections within the network are realized), centralization (extent to which a network is organized around its most central point), normalized degree centrality (extent to which all potential connections with an organization are realized), and normalized betweenness centrality (potential mediation or flow of information between organization in a network) (see full definitions of the network measures in Table 2). Table 3 provides normalized degree centrality (hereafter referred to as degree centrality) and normalized betweenness centrality scores (hereafter referred to as betweenness centrality) for the overall regional network and each of the nutrition working relationships.

**Table 3:** Normalized degree centrality and betweenness centrality for overall relationships and the nutrition actions

Organization†	Normalized degree centrality (%)					Normalized betweenness centrality (%)				
	**Whole network**	**Policy and legislation**	**Capacity development**	**Knowledge management**	**Implementation**	**Whole network**	**Policy and legislation**	**Capacity development**	**Knowledge management**	**Implementation**
A&T	12.9	11.6	4.7	9.3	7.0	1.1	1.5	1.8	0.1	0.2
ADB	7.0	4.7	4.7	4.7	4.7	0.5	0.2	0.1	0.1	0.6
AKDN	5.8	4.7	2.3	2.3	2.3	0.4	0.0	0.0	0.0	0.0
AKU_CWC	5.8	9.3	7.0	7.0	7.0	0.3	1.3	0.0	3.5	3.0
ANGOC	1.2	2.3	2.3	2.3	2.3	0.0	0.0	0.0	0.0	0.0
ARNEC	1.2	0.0	0.0	2.3	0.0	0.0	0.0	0.0	0.0	0.0
BMGF	22.1	14.0	7.0	14.0	11.6	6.3	3.8	1.4	0.4	0.8
BRAC	17.4	11.6	16.3	18.6	16.3	3.6	5.0	10.2	1.4	8.5
CivilSUN	11.6	16.3	11.6	14.0	7.0	5.6	8.3	5.9	1.2	3.3
COSATT	0.0	0.0	0.0	0.0	0.0	0.0	0.0	0.0	0.0	0.0
DFID	11.6	2.3	2.3	7.0	4.7	1.5	0.0	0.0	0.3	0.3
ECHO	10.5	0.0	0.0	7.0	2.3	0.4	0.0	0.0	0.1	0.0
EMRO	0.0	2.3	2.3	2.3	0.0	0.0	0.0	0.0	0.0	0.0
ENN	7.0	2.3	0.0	11.6	0.0	0.6	0.0	0.0	1.0	0.0
EUDEVCO	8.1	2.3	2.3	2.3	0.0	0.2	0.0	0.0	0.0	0.0
FAO	18.6	20.9	14.0	23.3	14.0	8.0	10.0	8.7	6.7	13.7
GAIN	12.8	14.0	14.0	18.6	11.6	1.7	4.0	9.6	3.1	4.4
HKI	12.8	9.3	4.7	14.0	9.3	1.2	1.4	0.5	0.6	4.3
HomeNet	0.0	0.0	0.0	0.0	0.0	0.0	0.0	0.0	0.0	0.0
IBFAN	3.5	2.3	2.3	2.3	0.0	0.0	0.0	0.0	0.0	0.0
IFPRI	18.6	9.3	4.7	34.9	11.6	3.1	3.3	0.1	9.0	1.6
IGN	8.1	2.3	2.3	4.7	9.3	0.2	0.0	0.0	0.0	5.5
ILO_DWT	1.2	0.0	0.0	0.0	0.0	0.0	0.0	0.0	0.0	0.0
LANSA	11.6	4.7	4.7	16.3	2.3	1.3	0.0	1.3	2.1	0.0
NI	11.6	2.3	0.0	9.3	7.0	1.3	0.0	0.0	0.6	0.8
SAARC	7.0	14.0	7.0	11.6	7.0	4.5	7.9	1.9	4.6	3.8
SAFANSI	23.3	20.9	16.3	32.6	14.0	9.1	14.5	12.9	14.0	6.1
SAIFRN	8.1	0.0	4.7	9.3	0.0	0.4	0.0	1.1	0.2	0.0
SAPLING	5.8	0.0	2.3	0.0	0.0	0.0	0.0	0.0	0.0	0.0
SAVE	14.0	0.0	0.0	0.0	0.0	1.4	0.0	0.0	0.0	0.0
SAWTEE	1.2	2.3	0.0	2.3	2.3	0.0	0.0	0.0	0.0	0.0
SDSN	0.0	0.0	0.0	0.0	0.0	0.0	0.0	0.0	0.0	0.0
SEARO	17.4	11.6	9.3	20.9	9.3	2.9	4.7	4.9	7.0	7.5
TATA	10.5	4.7	2.3	16.3	11.6	0.8	0.0	0.0	1.5	2.2
TCI	4.7	2.3	0.0	7.0	0.0	0.0	0.0	0.0	0.0	0.0
UNFPA	8.1	2.3	0.0	0.0	0.0	0.7	0.0	0.0	0.0	0.0
UNICEF	30.2	16.3	14.0	23.3	4.7	21.0	6.0	11.5	7.4	0.9
USAID	10.5	11.6	0.0	9.3	2.3	0.7	1.7	0.0	0.3	0.0
Water	9.3	0.0	0.0	0.0	0.0	1.2	0.0	0.0	0.0	0.0
WB	14.0	18.6	16.3	25.6	20.9	1.0	8.8	12.1	5.4	10.2
WFP	12.8	4.7	2.3	11.6	7.0	1.3	0.1	0.0	0.7	3.4
WRA	1.2	2.3	2.3	0.0	2.3	0.0	0.0	0.0	0.0	0.0
WSSCC	3.5	2.3	2.3	0.0	2.3	0.0	0.0	0.0	0.0	0.0

^**†**^See Table 1 for definitions of organizational acronyms

#### Overall regional network

**Fig. 1:**
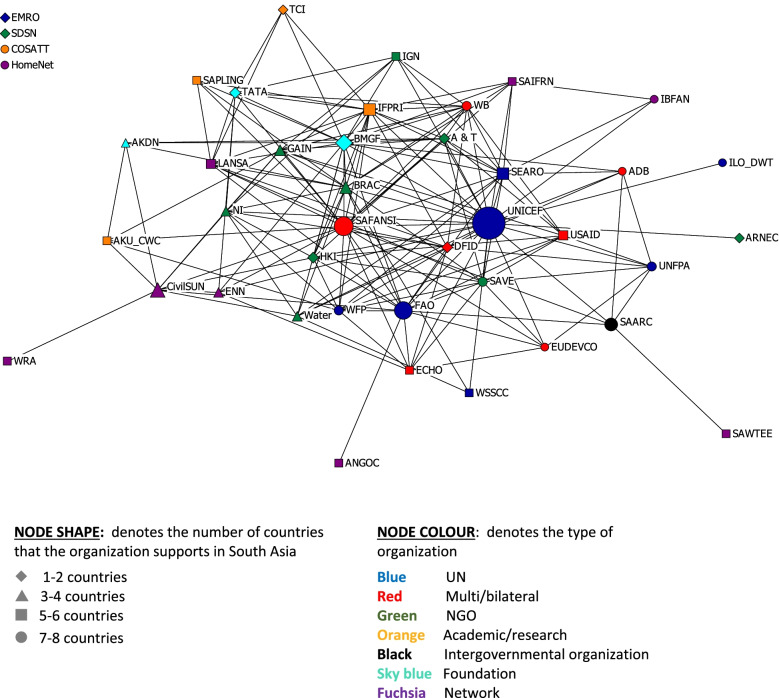
Confirmed relationships between regional-level organizations in South Asia, nodes sized by betweenness centrality. See Table 1 for definitions of organizational acronyms

**Fig. 2:**
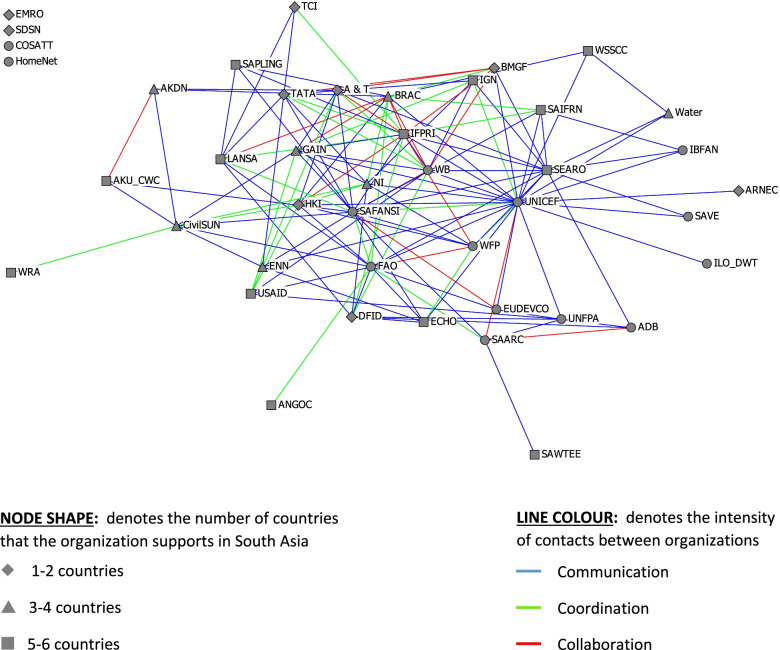
Confirmed intensity of working relationship between regional-level organizations in South Asia. See Table 1 for definitions of organizational acronyms

Figure 1 shows the confirmed relationships between organizations on any purpose (including but not specific to nutrition) in the two years prior to the study, and Fig. 2 shows the intensity of these relationships. The density of the overall regional network was 19.5% and the centralization score was 22.4%. There was a high degree of connectivity between most organizations in the overall network, especially those centrally located in the network. Organizations of the same or similar type appear to exhibit a pattern of clustering. For example, UN agencies were surrounded by donors, NGOs were clustered and connected to each other, and academic/research organizations were on the periphery. UNICEF had the highest number of connections, followed by SAFANSI, BMGF, FAO and IFPRI. The number of connections tends to be higher for organizations that worked in a larger number of South Asian countries. The nodes in Fig. 1 were sized by betweenness centrality which signifies the ability to connect organizations that do not have a direct channel to other organizations. UNICEF ROSA had the largest node, followed by SAFANSI, FAO, BMGF and CivilSUN.

Most of the relationships between organizations consisted of basic communication (the lowest level of relationship intensity), and there were only a few collaborative relationships (the highest level of relationship intensity) (Fig. 2). Coordination relationships were concentrated among NGOs and sub-networks.

#### Nutrition working relationship networks

To explore how the organizations interacted on each of the four nutrition working relationships, we assessed density, centralization, degree centrality and betweenness centrality. Table 3 provides degree centrality and betweenness centrality scores for each of the nutrition working relationships by organization.

Density was highest for the knowledge management network (9.5%), followed by the policy (6.3%), implementation (5.2%) and capacity development (4.5%) networks. The large difference between the density score for the whole network (19.5%) and nutrition working relationships indicates there was a gap in relationships between organizations that know each other and the work that they do together on nutrition. Centralization was highest for knowledge management (27.5%), followed by implementation (17.1%), policy (15.9%) and capacity development (12.8%). FAO, SAFANSI and WB were among the organizations with the top-five degree centrality scores for all nutrition working relationships that signified their potential brokerage positions in the network. Only SAFANSI had the top five betweenness centrality scores for all nutrition working relationships; FAO and WB were ranked in the top five for three of the four nutrition working relationships.

**Fig. 3:**
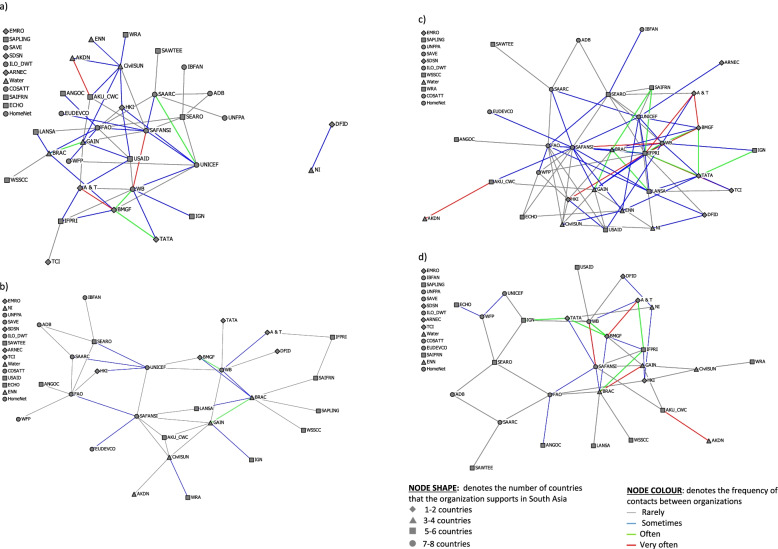
Confirmed frequency of interactions between regional-level organizations in South Asia on (**a**) nutrition policy, legislation, strategies or plans, (**b**) nutrition capacity development, (**c**) nutrition knowledge management and (**d**) implementation of nutrition interventions. See Table 1 for definitions of organizational acronyms

The nutrition policy, legislation, strategies and plans network (Fig. 3a) had four main centres of connectivity. SAFANSI seems to hold the central position, with the highest betweenness centrality (14.5%), and was connected to the next tier of organizations that could enable channels for communicating throughout the whole network (FAO, WB, SAARC and UNICEF). The central players operated in 7–8 countries, while those on the periphery worked in fewer countries. Most of the interactions over the last two years were listed as ‘rarely’ or ‘sometimes’. There were only four ‘often’ levels of interaction (SAARC-UNICEF, BMGF-TATA, BMGF-WB, BRAC-GAIN) and three ‘very often’ interactions (WB-SAFANSI, BMGF-A &T, AKDN-AKU_CWC).

The capacity development network for nutrition were dispersed and appear to have multiple clusters of different sets of players, mainly SAFANSI, World Bank, UNICEF and BRAC (Fig. 3b). BRAC, SAFANSI and WB were tied for the highest degree centrality (16.3%), and SAFANSI and WB also possessed the highest betweenness centrality (12.1%). Most of the organizations interacted only ‘rarely’ or ‘sometimes’. There were only two ‘often’ levels of engagement (GAIN-BRAC and BMGF-WB) and no connections were at the highest level of intensity (‘very often’).

The knowledge management network had the highest density score and thus the highest level of connectivity of all nutrition working relationships (Fig. 3c). IFPRI had the highest degree centrality (34.9) and was at the centre of the dense right side of the plot. On the left side, SAFANSI was at the centre and had the highest betweenness centrality (14.0%), followed by IFPRI (9.0%). The plot was shaped like a hub and spoke with SAFANSI and IFPRI having the capacity to create information and knowledge exchange among other organizations in the network. Most of the organizations worked together ‘rarely’ or ‘sometimes’ over the last two years. There were fewer ‘often’ and ‘very often’ interactions.

There were two visible clusters in the implementation network (Fig. 3d). Degree centrality scores were highest for WB (20.9%), followed by BRAC (16.3%), FAO and SAFANSI (both at 14.0%), which all fall on the right-hand side of the plot. The NGOs were near the donors and foundations, which most likely supported the NGOs’ implementation activities. The left side was sparely connected and dominated by UN agencies. FAO, WB and BRAC provided points of contact between the two clusters. There were many organizations on the periphery of the plot that connected with only one other organization and were at risk of disassociation from the network. Organizations in the left cluster of the network were mainly interacting ‘rarely’; only WFP-ECHO and WFP-UNICEF pairs ‘sometimes’ worked together on implementation. The right side of the plot was more active, with five pairs of organizations listing their interactions as ‘often’ and four as ‘very often’.

**Fig. 4:**
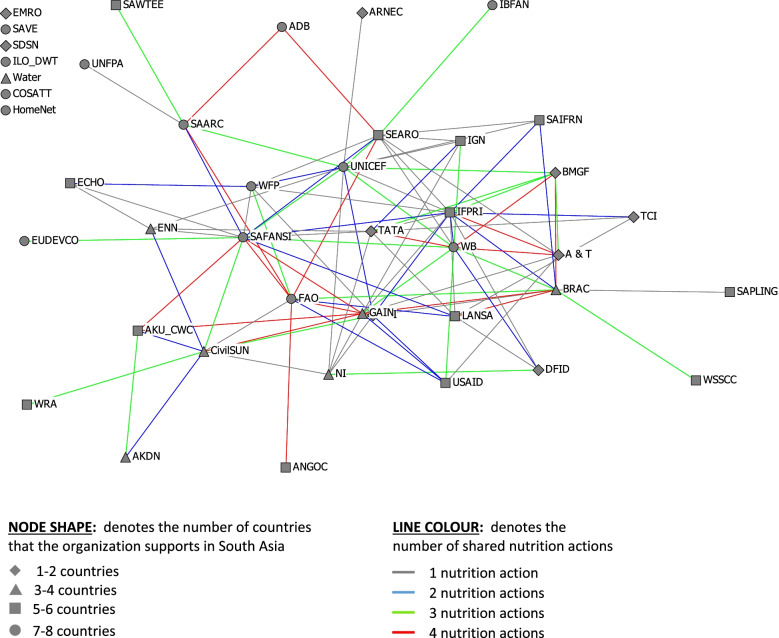
Confirmed multiplexity of relationships between regional-level organizations in South Asia. See Table 1 for definitions of organizational acronyms

A robust organizational network consists of multiplex relationships in which organizations have multiple types of relationships. Figure 4 depicts the multiplex network; the ties are colour coded by the number of nutrition working relationships that connect two organizations. There was a balance of different coloured lines signifying the four levels of multiplicity from one to four working relationships. Some organizations had stronger ties and worked together on three or four working relationships (green or red) while other organizations identified only one or two joint working relationships.

### Qualitative findings

All 26 targeted organizations participated in the semi-structured interviews (Table 1). These organizations included UN agencies (19%), multilateral and bilateral donors (8%), NGOs (27%), academic and research institutes (8%), an intergovernmental organization (4%), foundations (12%), and networks (23%).

#### Status of regional networks on nutrition

Organizational respondents agreed that South Asia did not have a strong experience of multi-organizational regional networks on nutrition, and there was a lack of viable regional mechanisms to support communication, coordination, and collaboration between organizations. One suggested reason was that nutrition was not considered a regional issue by governments or partners, unlike other cross-country issues such as human trafficking and natural disasters, and so it was not prioritized for regional cooperation.

Many respondents did not have any experience with multi-organizational regional networks for nutrition in South Asia, and some doubted that they exist in other regions. Those that did acknowledge their presence in South Asia perceived the existing structures to be weak or ineffectual, often operating in a manner that is “*diffuse and event like*” (multilateral donor), with insufficient rigour in ensuring long-term engagement and sustainability.

Existing platforms included the SUN Movement and the South Asia Association for Regional Cooperation (SAARC). Five countries in South Asia (Afghanistan, Bangladesh, Nepal, Pakistan, and Sri Lanka) and four states in India were members of the SUN Movement. There was no regional multi-stakeholder network for the SUN Movement in South Asia. However, there was two single-stakeholder regional networks in the Asia region: the Asia UN Network for SUN and the Asia Civil Societies Group for SUN. Some respondents viewed SUN as a mechanism within countries to rally stakeholders and sectors around nutrition and saw the potential to expand its influence at the regional level:

*For many countries [the SUN Movement] serves as a learning and accountability network. The questions we are asking whether we can get this at the regional level.* (Academic)

Others questioned the potential influence of SUN in the region, given three countries in South Asia were not members, including India, which is home to the majority of malnourished children.

In contrast, there was a strong belief that SAARC had the legitimacy to support intergovernmental cooperation in South Asia and could strengthen its leadership role on nutrition. In 2013, SAARC released its South Asia Regional Action Framework for Nutrition to guide member states on improving nutrition. Several respondents were unaware of the framework, and others doubted that it was being actively implemented or monitored. Some mentioned the recurrent difficulties that SAARC faces in convening member states due to geopolitical constraints in the region. However, most respondents recognized the untapped potential of SAARC:

*If it were possible for a [SAARC] unit to take the responsibility to coordinate for purpose of undernutrition … it would bring regional cooperation to a much stronger point.* (NGO)

*It would be a lot if SAARC could start to propel [evidence-based] discussions and encourage that way of thinking. So that the evidence base that exists starts to become part of policy discussion.* (Network)

*We are looking at how we can work together with SAARC to build a common commitment across member states to adopt policy and programme decisions to improve nutrition. We see when countries come together, they can learn and inspire each other …* (UN)

#### Factors influencing regional-level communication, coordination or collaboration on nutrition

The respondents identified several common factors that influence communication, coordination or collaboration on nutrition between organizations at the regional level. These were categorized into internal and external factors and presented with representative quotations in Table 4.

**Table 4:** Respondent perceptions on factors affecting organizational communication, coordination or collaboration at regional level

**INTERNAL FACTORS**	**Representative quotations**
**Organizational agendas and mandates**	“Every organization has its own agenda, very difficult to bring them on the same platform with a same vision.” (Network)
	“There are varied mandates, even in UN system, with fragmentation.” (Multilateral)
**Organizations’ visibility and branding**	“People are always worried about coming together on a common platform…as you want your own visibility – that’s the way you raise resources.” (Foundation)
	“Another big drawback to… regional [coordination] is that urge and instinct to have your logo everywhere, that I am the key driver everywhere of this program. The moment that you give that up, you can come together.” (Foundation)
**Organizations’ coverage of countries and location of regional hubs**	“We are working in [geographically dispersed] locations as a regional community. There is no real hub where we are physically together. Because there has been no physical contact and even electronic or online contact …discussions have not really taken place and it is rather at an ad hoc level that we are coming together.” (UN)
	“Different UN agencies recognize different geographic regions. For example, UNICEF has a regional office covering South Asia, while other UN agencies recognize a single Asia and Pacific region. For this reason, we may vary in our capacity to focus on the South Asia region.” (UN)
**Organizations’ human and financial resources to support regional cooperation**	“The kind of issues that become challenging is when it gets to what needs to be done and what resources are needed, who is there to respond to requests and engage with network conversations.” (Academic)
	“It’s somehow a challenge because each organization comes with a source of funding for them with so many strings attached. Flexibility is not there and that is where the donor money restricts you.” (Foundation)
	“We need staff to be completely dedicated to doing some of this work [on regional level coordination] … we need to commit for the human resource time.” (INGO)
**Existing agreements, history and trust with other organizations**	“In some instances where there is a global or regional MOU [Memorandum of Understanding]. This strengthens the opportunities because there is an agency interest in bringing organizations together.” (UN)
	What has worked is when we've had that common history [with another organization] and we are using that as a platform to build and strengthen those relationships. What we are missing is a knowledge of what opportunities exist. What is driving our limited coordination [at regional level] at the moment, is reaching out to where there is a history of collaboration globally.” (UN)
	“We tried to come together [with other organizations] in India and Bangladesh – takes lots of time to build trust and go together with common objective. There is an effort among partners to come together.” (Multilateral)
	“If you have like-minded individuals, then people see the benefits of working together and it can work.” (UN)
**Conflicts of interest among network members**	“Some of the more regionally focused meetings have been convened by the private sector and the companies we might not necessarily want to work closely with…We have concerns with partnering with some of these organizations.” (NGO)
	“Concept of coordination and collaboration is good but need policy for conflict of interest.” (Network)
**EXTERNAL FACTORS**	
**Regional geopolitical context**	“In South Asia region, [some] countries don’t enjoy good relations like India, Pakistan and Bangladesh…Civil society wants to collaborate with each other. We try to meet in Bangkok or other neutral places, but our countries do not want people to people contact.” (Network)
**Regional common narrative and platform for cooperation**	“There isn’t a coherent narrative that goes across nutrition to work in … [supporting a unified regional agenda].” (Foundation)
	“[We need] to develop a mechanism so that we are better coordinated and create an attitude of openness that we are working in tandem instead of in competition and in isolation.” (NGO)
**Donor expectations**	“In some instances, donors will also expect or look for organizations working together… that is another push mechanism for bringing organizations together.” (UN)

Many internal factors were expressed as constraints, indicating that organizations perceived more internal barriers than enablers to improving regional cooperation. The flexibility and willingness to work within regional partnerships and networks depended on an organization’s agenda and mandate, perceived threats to visibility and branding, coverage of countries in South Asia, location of the regional hub, human and financial resources, existing agreements, history and trust with other organizations, and perceived conflicts of interests among potential partnership or network members.

Some respondents stated that the dominance of the UN and World Bank and the difficulty in creating equal power relationships among organizations was a possible barrier to regional cooperation. Other external factors included the geopolitical context that hinders cooperation between some countries in the region, the absence of a unified common narrative and network for cooperation among regional-level organizations on nutrition, and donor expectations that may discourage or encourage partnerships and networks at the regional level.

#### Demand for regional networks on nutrition

Despite the challenges, there was a common desire to strengthen regional communication, coordination, and collaboration on nutrition. Respondents expressed concern that opportunities to work together were being missed, that “*The full potential of coordination among groups has not been realized*” (Foundation) and that the lack of cooperation at the regional level was “… *a contributing factor [in holding back progress on nutrition]*” (UN). They recognized the opportunities to make better use of organizational resources and achieve greater impact on nutrition but cautioned that any joint regional initiatives must lead to tangible results at the country level: “… *what will be value added of regional collaboration is action at the country level*” (Multilateral donor).

## Discussion

This study examined the structure and relationship dynamics between international organizations working in at least two countries or at regional level to improve maternal and child nutrition in South Asia. We found a high degree of connectivity between organizations in the overall regional network, but there were gaps in relationships between the many organizations that knew each other and the work they did together regionally on nutrition. Most organizations worked together only ‘rarely’ or ‘sometimes’ at regional level on nutrition and high-intensity (collaborative) working relationships were uncommon. Weak connections are a concern because they may make a network vulnerable to collapse.

However, a higher number of weak ties may allow for more diffusion of information and influence in contrast to a smaller number of strong ties [21].

Our study respondents recognized that opportunities to work together with a broader but less connected organizations on maternal and child nutrition were being missed and may contribute to the lack of progress in the region. They identified a range of internal and external factors that were perceived to undermine the nutrition working relationships, including organizations’ agenda and mandate, perceived threats to visibility and branding, coverage of countries in South Asia, location of the regional hub, human and financial resources, history, trust and power relations with other organizations, absence of a regional network for cooperation, and donor expectations. Complex interactions are a feature of networks because organizational autonomy encourages attention to one’s own perception of problems, solutions, and strategies [22, 23]. Such patterns of self-interest are common in many organizations that are bound to their own constituencies or stakeholders, including funders and regulators. Such organizations do not always believe that cooperation is in their organization’s best interest, especially when it means the agency’s managerial autonomy may be diminished and scarce resources must be shared [24].

A high degree of connectivity among most of the organizations in South Asia signifies a high level of potential information exchange and access to resources [25], which creates a strong foundation for further development and strengthening of the working relationships on nutrition. However, several challenges in the network structure and relationship patterns are evident.

First, organizations of the same type tended to cluster together, a pattern known as the homophily principle of networks, in which there is a tendency for organizations with similar characteristics to be connected [26]. This may mean they tend to engage with like-minded organizations, which could limit the opportunities to build connections across different organizational types and explore new ideas and novel approaches [13].

Second, a small number of UN agencies and multilateral organizations were central brokers in both the overall relationship and the nutrition working relationships (UNICEF, FAO, SAFANSI and WB). While these organizations operate in all South Asian countries and may more easily be activated to contribute to the network building process, they should ensure that they do not dominate the power relations within the network as less powerful partners may have more difficulty than others in advocating their interests and contributing to overall network effectiveness [18]. However, having a dominant core group may drive how a network develops by providing opportunities to transmit information, norms and values, and by building consensus on the critical tasks and goals to be accomplished by the network [27], which are pre‐cursor conditions for enhancing network performance.

Third, the low intensity and frequency of connections between organizations signals the need to establish more frequent points of contact to strengthen relationships and build platforms to coordinate and collaborate on nutrition. High intensity working relationships tend to arise only with frequent relations of extended duration [28]. In addition, there was no formal organized structure for activating joint engagement on key nutrition issues between organizations in the network, which may have contributed to their lack of connectivity. Positive relationships and more frequent interactions enable coordination and safeguard exchanges [29]. If prior relationships do not exist, then partnerships are likely to emerge more incrementally and begin with small, informal transactions that do not require much trust [30]. Collaboration partners build trust by sharing information and knowledge and demonstrating competency, good intentions, and follow through; conversely, failure to follow through and unilateral action undermine trust [18]. High levels of pre-existing interorganizational trust increases the probability that a less formal and hierarchical, and thus less costly, mode of governance is chosen over a more formal one [31].

Fourth, several organizations were ‘isolates’ and left out of the nutrition working relationships. These organizations tended to operate in only a few South Asian countries and may have less motivation or capacity to engage in regional-level relationships with other organizations. Prior research has demonstrated that non-governmental actors tend to be disconnected even though they need contact with others to broaden their legitimacy and potential impact [32]. It is important for networks to include organizations that are beyond their traditional base and reflect different value orientations to broaden support and influence [33]. Over time, successful networks will expand their linkages with organizations on the periphery and mature into an integrated dynamic whole that allows the essential flow of information, resources, and services to positively impact their countries and communities [24]. However, networks can become inefficient if they comprise too many connections [27, 34] and so knowledge exchange and strategic engagement of these peripheral players rather than formal connections may be sufficient.

Knowledge transfer across organizational boundaries is a central issue in the organizational literature as people access knowledge across geographic space via their network ties [35, 36]. We found that density and degree centrality were higher for the knowledge management network than for the other nutrition working relationships, suggesting it may be an important starting point for building stronger working relationships on nutrition at the regional level in South Asia. High density is likely to be advantageous for knowledge management because more ties mean there are more paths for knowledge to flow between organizations [37]. However, care is needed to ensure the network does not become too dense, creating unwanted complexities in management and coordination of network activities [35].

Transnational networks have existed for a long time but their number, size, professionalism, and the density and complexity of their international linkages have grown dramatically in recent decades [32]. Regional networks are strategically positioned as a ‘bridge or connector’ between the global and country levels and can mobilize international and regional organizations around a set of mutual goals in a common set of countries. Countries rarely develop policies in isolation from other countries, but rather acquire and promote reforms via a transnational process of policy diffusion [38]. Regional networks can help in this process by expanding the opportunities to facilitate knowledge exchange between global and country levels and between countries, advocate more persuasively according to country and regional contexts, and ensure capacity gaps are addressed more comprehensively [8, 32].

This is the first study, to our knowledge, that has used Social Network Analysis to examine the relationships between international organizations working at regional level on maternal and child nutrition. The findings of this study come at a time when there is increased interest and commitment from the global SUN Movement, international organizations, SAARC, and member states in South Asia to strengthen the role of regional networks in supporting countries to achieve national and global targets on nutrition [8, 9, 39]. The study adds to the growing body of research that demonstrates how ONA or Social Network Analysis can be used to assess connectivity patterns within networks and the behaviour of member organizations [14, 16], and guide the process to build more effective regional networks for multisector and multistakeholder engagement. This approach can be applied in other regions seeking to introduce or strengthen regional-level collaboration between organizations on nutrition and other areas of development.

Further research is needed to understand what shapes the effectiveness of regional nutrition networks, including their ability to influence policy or programme decisions and resources for nutrition at country level. Following the completion of this study, UNICEF initiated the formation of the Network for Improved Nutrition in South Asia (NINSA), which brings together UN agencies, donors, civil society, foundations and networks operating at regional or subregional level in South Asia, to improve communication, coordination and collaboration on maternal and child nutrition. Additional ONA studies can be used to track network measures over time to assess the changes in network structure and working relationships among NINSA member organizations.

Our study has several limitations. ONA and qualitative methods do not generate results that can ascribe causality or be generalized to other settings. The use of ONA to assess multistakeholder collaboration is a new area of work, particularly at the regional level, with no established standards of what constitutes a strong or a weak network. The selection of regional organizations and respondents was based on suggestions from key informants and internet searches and may have excluded lessor known and connected organizations. It is possible that some respondents did not have full knowledge of organizational interactions at the regional level. However, we mitigated this by allowing respondents to nominate better-informed colleagues from their own organization. The lead researcher was employed by UNICEF ROSA, and controls were put into place to reduce potential bias: no UNICEF staff were present during the interviews with other organizations and the data were accessible to only the lead researcher and her research analyst. Lastly, we did not include country or global level stakeholders in the study, and so the findings only reflect the perspectives of regional-level organizations.

## Conclusions

We find that opportunities are being missed for international organizations to work together on maternal and child nutrition at the regional level in South Asia. Despite a high degree of connectivity between organizations in the overall regional network, working relationships on maternal and child nutrition were characterized by low intensity and low frequency of contact. However, there was high demand among international organizations to remedy this situation and strengthen regional-level communication, coordination and collaboration on nutrition in ways that will have a meaningful impact on countries. The global SUN movement is in a key position to leverage greater attention to regional nutrition networks because of its role in strengthening the coordination architecture on nutrition. The effectiveness of regional nutrition networks in influencing policy or programme decisions and resources for nutrition at country level should be further explored.

## Data Availability

The datasets analysed during the current study are available from the corresponding author upon reasonable request.

## Abbreviations

NGO: Non-government organization
NINSA: Network for Improved Nutrition in South Asia
ONA: Organizational Network Analysis
ROSA: Regional Office for South Asia
SUN: Scaling Up Nutrition
UN: United Nations
UNICEF: United Nations Children’s Fund
